# Modern contraceptive utilization and associated factors among married pastoralist women in Bale eco-region, Bale Zone, South East Ethiopia

**DOI:** 10.1186/s12913-017-2115-5

**Published:** 2017-03-14

**Authors:** Semere Sileshi Belda, Mekonnen Tegegne Haile, Abulie Takele Melku, Abdurehaman Kalu Tololu

**Affiliations:** 1 Department of Public Health, Madda Walabu University, Goba Referral Hospital, P.O.Box 302, Bale-Goba, Ethiopia; 2 Department of Nursing, Madda Walabu University, Goba Referral Hospital, Bale Goba, Ethiopia

**Keywords:** Modern contraceptive utilization, Associated factors, Pastoralist women, Bale eco-region

## Abstract

**Background:**

Women who live in remote rural areas encounter different challenges against contraception and often deny the use of modern contraceptive methods. The predictors of modern contraceptive utilization by pastoralist women in the Bale eco-region could be specific and are not well known. Therefore, this study aims to assess modern contraceptive utilization and its determinants among married pastoralist women in Bale eco-region, Oromia regional state, South East Ethiopia.

**Methods:**

A community-based cross-sectional study was conducted from 20th November 2015 to 30th February 2016. A structured questionnaire was used to interview 549 married pastoralist women who were selected by multistage sampling technique. The data were analyzed by SPSS - 21 software, multivariate logistic regression analysis was used to identify predictors of modern contraceptive use at (*P*-value <0.05), and odds ratios with 95% confidence interval were used to assess the strength of associations between variables.

**Results:**

The current modern contraceptive method use by married pastoralist women was (20.8%). Among the total users, (78.1%) use the injectable method. The common reasons for non-use of modern contraceptive methods includes: religious-opposition (55.9%), desire for more children (28.3%), fear of side effects (25.5%), and husband’s opposition (17.5%). Couple discussion (AOR = 4.63, 95%CI: 2.15, 9.98), perceived husband’s approval (AOR = 8.00, 95% CI: 3.52, 18.19), discussion with health extension worker (AOR = 5.99, 95% CI: 1.81, 19.85), and perceived cultural acceptability (AOR = 2.10, 95% CI: 1.09, 4.03) were the independent predictors of modern contraceptive use by married pastoralist women in Bale eco-region.

**Conclusion:**

The study identified lower modern contraceptive method utilization by pastoralist women, and the majority of the contraceptive users rely on short- acting contraceptive methods. The uncomplimentary perceptions towards religious and cultural acceptability of modern contraceptive method were among the major reasons for lesser utilization of the methods. Family planning programs should be tailored to actively involve pastoralist women, husbands, and religious leaders in pastoralist communities.

**Electronic supplementary material:**

The online version of this article (doi:10.1186/s12913-017-2115-5) contains supplementary material, which is available to authorized users.

## Background

In 2016, world population stood on 7.4 billion. Africa accounts to more than 1.2 billion of the world population of which, Ethiopia, the second populous country in Africa contributes 101.7 million people. The average total fertility rate (TFR) worldwide ranges from 1.7 children per women in more developed countries to 4.3 in the least developed nations [1]. Evidence of fertility transition in developing countries reported that fertility and projected population growth are much higher in sub-Saharan Africa than in any other region of the world [2, 3]. TFR in Ethiopia is 4.2 children per women. This puts Ethiopia among countries with the highest fertility rates in the world [1, 4, 5]. The country also has a young population mainly due to the disproportionately high fertility rate among rural women who give birth to nearly three more children during their reproductive years than their urban counterparts [6, 7].

For fertilities to fall to those low levels, the increased use of modern contraceptive methods plays a significant role especially in the less developed countries including Ethiopia [1]. Besides its effect on fertility reduction, the use of modern contraceptive methods has a clear effect on the health of women, children, and families. Globally, contraceptives help to prevent an estimated 2.7 million infant deaths and the loss of 60 million of healthy life in a year [8]. It averts (32%) of all maternal deaths and nearly (10%) of childhood deaths and has the potential to reduce poverty and hunger especially in developing countries [9].

In the past decade, considerable efforts have been made by the government of Ethiopia and various local and international partners to expand family planning programs and services through building health infrastructure and the introduction of the health extension package (HEP). At present, although the national modern contraceptive use increased substantially, from (15%) in 2005 to nearly (42%) in 2014 [6, 7], the utilization is not consistent among the different segments of the community; for example, the highest contraceptive utilization rate was observed in major cities like Addis Ababa (57%), while the lowest (1.6%) and (13%) were reported in the Ethiopian Somali and Afar pastoralist regions respectively [7, 10]. And, such inconsistencies in contraceptive use among and within regions, and the lower utilization by communities who live in difficult terrain has been challenging Ethiopia who pledged during the 2012 London summit to uphold the rights of all Ethiopians to access voluntary family planning with special attention to serving isolated communities [11].

Though Health Extension Workers (HEWs) who provide modern contraceptive methods are assigned to the doorstep in rural kebeles, the utilization of modern contraceptive methods in pastoralist areas remained very low. This could be due to the reality that factors which influence modern contraceptive utilization by pastoralist women are multifaceted and challenging, which results in a continued higher fertility rate and unwanted pregnancy particularly in pastoralist communities which intern affects the health of mothers and children [5, 10]. Though several studies reported that the use of contraceptive service is determined by socio-demographic, socio-cultural, socio-economic, and the source of family planning information and service [6, 10, 12–17], evidences on the predictors of modern contraceptive method utilization are scarce and not well known in the context of pastoralist women in the study area. Therefore the current study was conducted with the intent to assess modern contraceptive method utilization and its determinants among married women in pastoralist communities of the Bale Eco-region (BER); so that the study findings will contribute to the development of context-specific strategies and family planning programs in pastoralist communities.

## Methods

### Study area and period

**Fig. 1:**
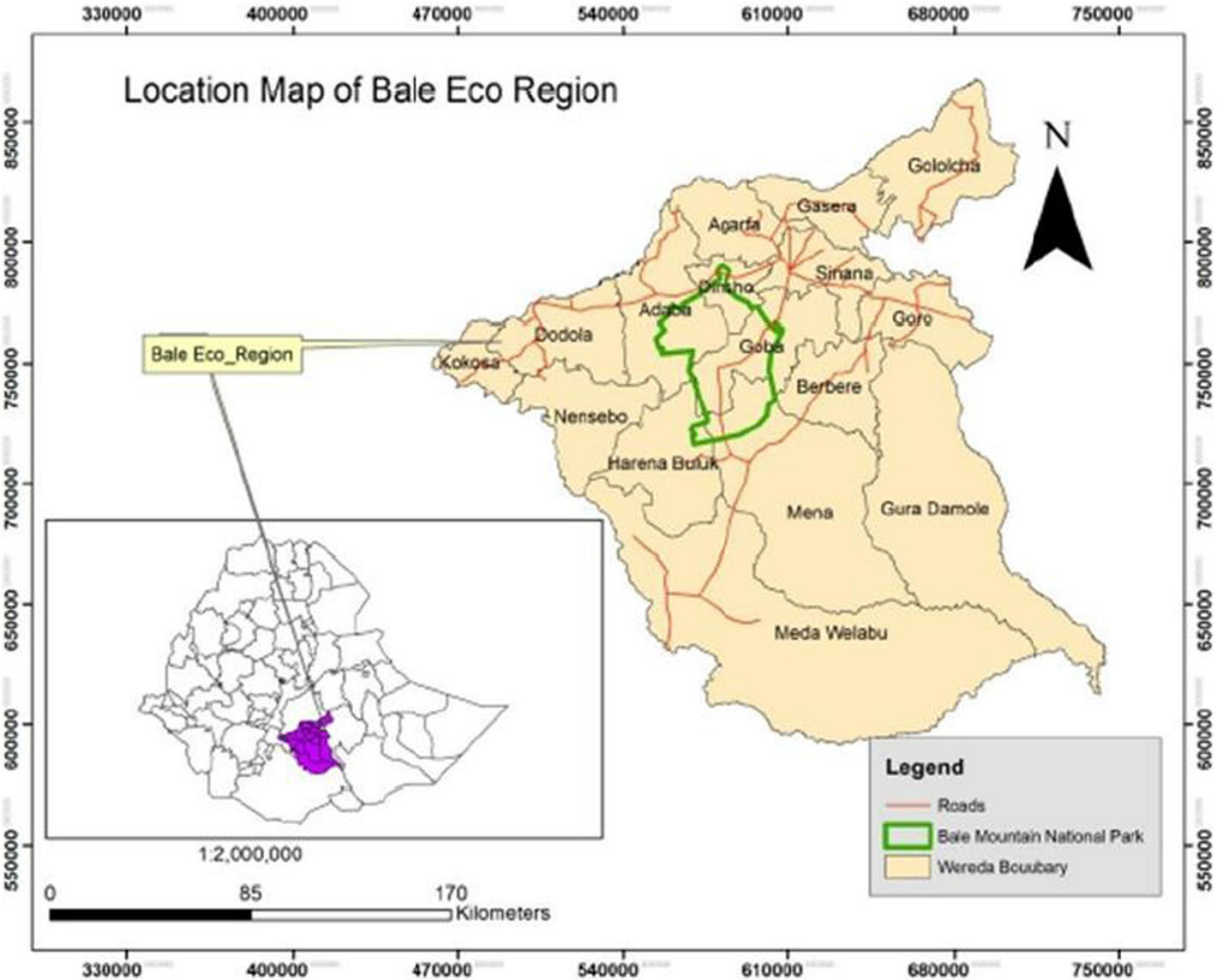
Bale Eco-Region indicating the Woreda boundaries and Bale Mountains National Park (BMNP) boundary, South East Ethiopia. (Map Adapted from SHARE Bale Eco region project document)

The study was conducted from November 20/2015 to February 30/2016 in pastoralist districts of the BER, Oromia region, Southeast Ethiopia. BER has 16 districts; where 12 of the districts are located in Bale zone and the other four districts are located in West Arsi zone (Fig. 1). Out of the 16 districts of the BER, only five (Madda Walabu, Dello Mena, Berbere, Gura Damole, and Harena Buluk districts) are pastoral districts and all of the pastoralist districts are geographically located in Bale zone.

The current study was conducted in Dello Mena, Harena Buluk and Madda Walabu districts. The main town, “Mena” of Dello Mena district, “Angetu” of Harena Buluk district and “Bidre” of Madda Walabu district are located at 555 km, 577 km and 626 km respectively in the South-east direction from Addis Ababa (Capital city of Ethiopia). The estimated total population was 114,813, 99,138 and 118,673 for Dello Mena, Harena Buluk and Madda Walabu districts respectively [4]. There is 1 hospital, 15 Health centres, and 45 Health posts in the study districts that are owned by the government. Family planning and maternal health services are provided free of charge in all public health facilities located in the study districts. Modern contraceptive methods in the form of Injection, Pills, Implants, Male condoms and counselling on Lactational Amenorrhoea Method (LAM) are available in the health posts. In addition to the contraceptive methods available at the health posts, the Intra Uterine Devices (IUDs) are available in the health centres and Hospital, and permanent contraceptive methods (surgical sterilization) are available in the Hospital.

### Study design and study population

A community based cross-sectional study design was used. The source population were all married pastoralist women (15–49 years) who live in pastoralist districts of the BER. The study population were all married pastoralist women (15–49 years) in the sampled kebeles.

### Sample size and sampling technique

The sample size was calculated using a single proportion sample size determination formula with the assumptions of a (22%) modern contraceptive utilization rate by married rural women in Oromia region [12], a 95% confidence level and absolute precision of 5%. The calculated sample size was 264 married pastoralist women (15–49 years). However, with a further assumption of a 10% none response rate and an adjustment for a design effect of two, the final calculated sample size for the study was 580 married pastoralist women (15–49 years).

A multistage stage sampling technique was employed to select the study units (married women of 15–49 years old). Initially three pastoralist districts (Dello Mena, Harena Buluk and Madda Walabu) were selected among the five pastoralist districts of the BER. There were ten pastoralist kebeles in Dello Mena district; nine pastoralist kebeles in Harena Buluk district and 12 pastoralist kebeles in Madda Walabu district. From each of the three selected districts, two kebeles (a total of 6 kebeles) were randomly selected for the study. Nanigadera and Melkamana kebeles from Dello Mena district; Mekanagobele and Anole kebeles from Harena Buluk district and Horakore and Medda kebeles from Madda Walabu district were the selected kebeles where the sample was drawn through a proportional allocation of the sample size based on the number of households in each selected kebele (Table 1). A list of households was obtained from the respective kebele administration offices and used as a sampling frame, and then households were selected using systematic random sampling method. For each selected kebele, the sampling interval “k” was determined (k = N/n) and the first interviewed household was identified using a lottery method among the households in the first sampling interval “K1”. The data collectors listed the sampled households and with the local guides directly gone to the selected households and conducted the interview. Further information on the study participants are provided in Additional file 1.

**Table 1:** Sample size distribution among sampled Kebeles and Districts, Bale eco-region, Southeast Ethiopia, April 2016

Name of sampled district	Name of sampled Kebele	Total number of household	Total sample
Dello Mena	Melkamana	680	102
	Nanigadera	707	106
Harena Buluk	Anole	620	93
	Mekanagobele	700	105
Madda Walabu	Medda	653	98
	Horakore	507	76
Total		3867	580

### Data quality control

Before the actual data collection, a 2 days training was given to the data collectors and the supervisors. The training focused on the objective of the study, the study population, the sampling procedure, the inclusion and exclusion criteria, the data collection tool (going through each question), interview techniques, data handling and storage. After the training, a pre-test was done by selecting 30 married pastoralist women in Melkarba kebele (a neighbouring pastoralist kebele in Harena Buluk district) and preliminary corrections were made on the questioner based on the findings obtained from the pre-test. Eight data collectors collected the data using a face to face interview. The principal investigator and the co-authors supervised the whole data collection process. After the data collectors filled the questioner, the supervisors reviewed it to ensure its completeness. Close supervision and early correction of errors were made during the data collection period.

### Data collection instrument and procedures

A structured and pre-tested questionnaire was prepared first in English and then translated into Afan Oromo (local language) by a native Afan Oromo speaker. Eight data collectors (diploma graduate nurses) who had previous experience of data collection collected the data. Eight local guides were recruited to travel with the data collectors and guide the boundary of the selected kebeles during the data collection period.

### Study variables

Modern contraceptive utilization was the main outcome variable (dependent variable) of the current study which was dichotomized in to modern contraceptive users and non-users. Users in our study were those pastoralist women or whose husband were using one of the modern contraceptive methods (oral contraceptive pill, injectables, implants, IUDs, condom, sterilization, or lactational amenorrhoea method) during the data collection time, and non-users were those women who or whose husband was not using modern contraceptive method during the data collection time. The independent variables include; socio-demographic characteristics, obstetric characteristics, contraceptive awareness, respondent’s perception, fertility desire, intension to use contraceptive, and communication with service provider. Information on possession of household assets like: livestock (number of cattle, goats/sheeps, camel etc.); amount of farm land; and possession of materials like radio, telephone, motorbike, etc. in the household were gathered to determine socio economic status (SES) or wealth index of the respondents as a composite measure of the household assets. So that a relative SES was assigned to each respondent’s household in quintiles (i.e. poorest, poorer, middle, richer, and richest) using Principal Component Analysis (PCA).

### Data processing and analysis

The data were checked for completeness and consistencies during the data collection, and then cleaned, coded and entered into a Statistical Package for Social Scientists (SPSS) windows version 21 computer software for analysis. Frequency checks were made for each study variable and further cleaning and cross checks were made to ensure the consistency of the study variables among respondents. Descriptive analysis was made and measures of central tendency were determined. Logistic regression models were applied to assess the presence of an association between the dependent variable (modern contraceptive use) and the independent variables at (*P* < 0.05). Odds ratios with 95% confidence interval were described in detail for explanatory variables. Findings related to the study objectives were presented in text, graph, and tables.

## Results

### Socio-demographic characteristics

Five hundred forty nine pastoralist women were successfully interviewed among the sampled 580 married women yielding a response rate of (94.7%). However, we were not able to interview 31 respondents due to their non-presence despite the data collectors repeated attempt to interview them. The mean age (±Standard Deviation) of the respondents was 26.9 (±7.6) years.

Nearly all (99.5%) of the study participants were Muslim and the other (0.5%) were non-Muslims by religion. Concerning the ethnic composition; the great majority (89.1%) of the respondents were Oromo and the other (9.0%) of the respondents were from Somali ethnic group. Regarding school enrolment; the majority (71.4%) of the respondents had no formal education, followed by (24.0%) with a primary level of education and the other (4.6%) had some secondary or post-secondary level of education. About (28.1%) and (18%) of the husbands had a primary and secondary or post-secondary level of education respectively. Regarding the assessment of marital form or the practice of having more than one wife (polygamy); (27.2%) of the respondents reported that their husbands have more than one wife or are in polygamous marriage (Table 2).

**Table 2:** Socio demographic characteristics of study participants, Bale eco-region, Bale Zone, South east Ethiopia, April 2016 (*n* = 549)

Variable	Category	Frequency	Percent
Respondents age	15–19 years	81	14.80
	20–24 years	147	26.80
	25–29 years	133	24.20
	30–34 years	83	15.10
	35–39 years	54	9.80
	40–44 years	33	6.00
	45–49 years	18	3.30
	Mean age(±SD)	26.9 (±7.6)	
Respondents religion	Muslim	546	99.50
	Orthodox	3	0.500
Respondents ethnicity	Oromo	489	89.10
	Somalie	55	10.00
	Other (Amahara & Sidama)	5	0.90
Respondents educational status (highest level of schooling)	No formal education	392	71.40
	Grade 1–6	132	24.00
	Grade 7 and above	25	4.60
Age of respondent’s husband (*n* = 532)	<= 24 years	39	7.30
	25–34 years	223	41.90
	>34 years	270	50.80
Educational status of respondent’s husband	No formal education	296	53.90
	Grade 1–6	154	28.10
	Grade 7 and above	99	18.00
Marital form of the respondent’s husband (*n* = 548)	Polygamous	149	27.20
	Monogamous	399	72.80
Number of wives (for Polygamous marriage) (*n* = 149)	2	121	22.10
	> = 3	28	5.10
Family size (people living in respondent’s household)	<= 4 people	216	39.30
	5–8 people	278	50.70
	> = 9 people	55	10.00
Availability of radio in respondent’s household	Yes	205	37.30
	No	344	67.70
Availability of mobile phone in respondent’s household	Yes	242	44.10
	No	307	55.90
Household Socio Economic Status Index quintiles	Lowest	115	20.90
	Lower	108	19.70
	Middle	103	18.80
	Higher	113	20.60
	Highest	110	20.00

### Obstetric characteristics of respondents

A large proportion (41.6%) of the study subjects were married the first time at or before 15 years of age and another (32.1%) were married by age 16 to 17 years. More than half (51.7%) of the respondents had more than four pregnancies, and (9.3%) of the respondents had a history of abortion (induced and/or spontaneous), and about (18.8%) of the respondents had child death experience. Some (9.8%) of the respondents desire to have an additional child within 2 years period, while significant proportion (67.7%) of the respondents want to delay or postpone the next childbirth for at least 2 years and the another (22.5%) of the respondents reported they do not need another child (Table 3).

**Table 3:** Obstetric characteristics of the study participants, Bale eco-region, Bale Zone, South east Ethiopia, April 2016 (*n* = 549)

Variables	Category	Frequency	Percent
Age of respondent at marriage (*n* = 543)	<= 15 years	226	41.6
	16–17 years	174	32.1
	> = 18 years	143	26.3
	Mean age at marriage(±SD)	16.3 (±1.9)	
Number of pregnancies	<4 pregnancies	265	48.3
	4–7 Pregnancies	201	36.6
	> = 8 Pregnancies	83	15.1
Number of deliveries	<4 births	277	50.5
	4–7 births	200	36.4
	> = 8 births	72	13.1
History of abortion (induced and/or spontaneous)	Yes	51	9.3
	No	498	90.7
Child loss experience	Yes	103	18.8
	No	446	81.2
Current pregnancy	Yes	72	13.1
	No	477	86.9
Status of current pregnancy (*n* = 72)	Planned	60	83.3
	Not planned	12	16.7
Number of children alive	0	34	6.2
	1–3	233	42.4
	4–7	209	38.1
	8+	73	13.3
Desire for more children	<2 years	54	9.8
	After 2 years	371	67.7
	Want no more	123	22.5
Desired number of additional children	0	117	25.7
	1–2	88	19.3
	3–5	191	41.9
	>5	60	13.1
Visited by HEW^*^ (in the last 12 month)	Yes	376	68.5
	No	173	31.5
FP Discussion with HEW^*^ (*n* = 376)	Discussed	306	81.4
	Not discussed	70	18.6
Proximity to healthcare facility	<15 min	300	54.6
	16–30min	166	30.2
	> = 30 min	83	15.1

HEW*-Health extension workers

### Knowledge of contraceptive methods

An overwhelming proportion (95.8%) of the respondents had the awareness about family planning methods, and (95.3%) of them mentioned at least one modern contraceptive method; oral contraceptive pills were the most commonly known methods mentioned by (91.3%) of the respondents, followed by injectable methods, implants and lactational amenorrhea methods which were mentioned by (88.3, 47.7 and 44.3%) of the respondents respectively. While the IUDs, Female, and Male sterilization methods were the least known methods which were mentioned only by (15.8, 3.85 and 2.4%) of the pastoralist women respectively. The main sources of contraceptive information include; HEW reported by (89.9%) of the respondents, followed by friends or neighbours (28.1%), other health workers (24.2%) and radio which was reported by (22.1%) of the respondents.

### Current modern contraceptive method utilization

The current utilization of modern contraceptive methods by married pastoralist women was (20.8%). Among the women who are currently using a modern contraceptive method; (78.1%) were using the injectable method, (9.6%) were using the oral contraceptive pills, (7.9%) were using implants, and (4.4%) were using the lactational amenorrhea method. However, none of the participants reported the use of the more effective long-acting modern contraceptive methods like the IUDs and the male and female sterilization methods. And another (5.8%) women reported the use of a traditional method (periodic abstinence) as a method to prevent unwanted pregnancy.

**Fig. 2:**
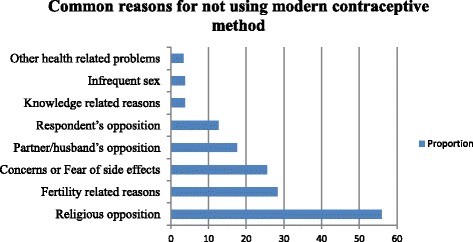
Reasons for not using modern contraceptive methods (*n* = 435) by pastoralist women in Bale eco-region, Bale Zone, South east Ethiopia, April, 2016

Those pastoralist women who were not using any modern contraceptive method during the survey (*n* = 435) were asked about the reasons for not using the modern contraceptive method. The main reasons for not using modern contraceptive were asked and recorded by the interviewers; hence analyzed based on multi-response questions (i.e., a woman can give more than one reason for not using any contraceptive method.) Accordingly more than half of the respondents (55.9%) reported religious opposition as the reason for non-use, (17.5%) partner (husband’s) opposition, (12.6%) respondent’s opposition, (28.3%) due to fertility-related reasons and (25.5%) concerns or fear of side effects (Fig. 2).

### Respondent’s perception about modern contraceptives

Among the total pastoralist women interviewed, perceptions about modern contraceptive methods and pregnancy spacing were as follows. Only (44.3%) of the women perceived that modern contraceptive is acceptable by the rest of the community members, (30.6%) of the interviewed women perceived that their husband do not oppose the use of modern contraceptive method and while great proportion (92.4%) of them perceived that the use of the modern contraceptive methods is not supported by religious leaders. Although, the majority (62.4%) of the respondents believe that couple discussion is useful to make a joint decision regarding the number and spacing of children in a family; nearly (70%) of the pastoralist women never discussed the issue of family planning and pregnancy spacing with their husbands (Table 4).

**Table 4:** Respondent’s perceptions about contraceptive methods, Bale eco-region, Bale Zone, South East Ethiopia, April, 2016 (*n* = 549)

Variable	Category	Frequency	Percent
Do you think FP is important to the wellbeing children and the family?	Yes	373	67.9
	No/not sure	176	32.1
Do you think FP is culturally accepted by the community?	Yes	243	44.3
	No/not sure	306	55.7
Do you think religious leaders support FP use? (*n* = 544)	Yes	40	7.6
	No	504	92.4
Who is responsible to make decisions about number of children in a family?	Wife	62	11.3
	Husband	144	26.3
	Both husband and wife	342	62.4
Have you ever discussed FP with your husband in the last 12 months?	Yes	165	30.1
	No	384	69.9
Do you think your Husband knows about FP and its benefits?	Yes	315	57.4
	No	234	42.6
Do you think your husband approves the use of modern contraceptive methods?	Yes	167	30.6
	No	379	69.4

### Factors associated with modern contraceptive use

Bivariate and multivariate logistic regression models were fitted to determine the presence of an association between the dependent variable (modern contraceptive utilization by married pastoralist women) and the independent variables at (*P* < 0.05) level of significance.

Those variables which had a statistically significant association (*P*-value ≤0.05) with modern contraceptive utilization in the bivariate analysis were hired for multiple logistic regression analysis. The findings from the multiple logistic regression analysis revealed that couple discussion, respondent’s perception about husband’s approval of modern contraceptive use, respondent’s discussion with HEW, and the desired time for additional children, and respondent’s perception about cultural acceptability of modern contraceptives were the independent predictors of modern contraceptive utilization.

Pastoralist women who discussed with their husbands about the issue of modern contraceptives and/or spacing the age between their children during the 12 months period before the survey were more than four and half times (AOR = 4.63, 95% CI: 2.15, 9.98) more likely to use modern contraceptives than those women who didn’t discussed with their husbands. Apart from the couple discussion, women’s perception about her husband’s view and approval of modern contraceptives had an independent effect (*p* < 0.001) on her modern contraceptives method utilization. Hence the odds of modern contraceptives method utilization among women who perceived that her husband supports the use of modern contraceptive was eight times (AOR = 8.00, 95% CI: 3.52, 18.19) higher than those women who do not perceive that her husband supports the use of modern contraceptives.

The other independent predictor which had a very strong association (*p* <0.01) with pastoralist women’s utilization of modern contraceptives services was the discussions made with a HEW in the last 12 months. Those women who discussed modern contraceptive with a HEW in the 12 months before the study period were 5.99 times (AOR = 5.99, 95% CI: 1.81, 19.85) more likely to use a modern contraceptive method than those women who didn’t discuss with a HEW. The output from the multivariate analysis revealed the presence of statistical association (*p* < 0.05) between a women’s desire to get an additional child and her use of modern contraceptive methods. The current study also reported that the odds of MC utilization were about 7.13 times (AOR = *7.13, 95% CI: 1.53, 33.30)* higher for women who want another child after 2 years than those women who desire to have the next child in less than 2 years’ time. The perception of the women about the community’s opinion or cultural acceptability towards family planning use had an independent effect on the women’s MC utilization (*p* < 0.05). The odds of modern contraceptive use among those pastoralist women who perceived that the use of modern contraceptives is culturally accepted by the majority of the community members was more than twice (AOR = 2.10, 95% CI: 1.09, 4.03) higher than those women who perceived that MC is not culturally acceptable (Table 5).

**Table 5:** Factors associated with modern contraceptive utilization among married pastoralist women in Bale eco-region, Bale Zone, South East Ethiopia, April, 2016

Variable	Modern contraceptive utilization		COR (95% CI)	AOR (95% CI)
	Non-users	Users		
	Frequency (%)	Frequency (%)		
Respondent’s age				
15–24 years	175(76.8)	53(23.2)	2.14 (1.11, 4.14)	2.37(0.65, 8.73)
25–34 years	168(77.8)	48(22.2)	2.02(1.04, 3.93)	1.92(0.66, 5.58)
35–49 years	92(87.6)	13(12.4)	ref	ref
Respondent’s educational status				
No formal education	326 (83.2)	66 (16.8)	ref	ref
Primary level education	100 (75.8)	32 (24.2)	1.58(0.98, 2.55)	1.51(0.63, 3.62)
Secondary level and above	9 (36.0)	16 (64.0)	8.78 (3.72, 20.72)	3.75(0.83, 16.88)
Educational status of respondent’s husband				
No formal education	243 (82.1)	53 (17.9)	ref	ref
Primary level education	139 (90.3)	15 (9.7)	0.50 (0.27, 0.91)	0.52(0.21, 1.28)
Secondary level and above	53 (53.3)	46 (46.5)	3.98 (2.43, 6.52)	1.66(0.66, 4.17)
Husbands age				
< = 24 years	31(79.5)	8(20.5)	Ref	Ref
25–34 years	170(76.2)	53(23.8)	1.21(0.52, 2.79)	1.94(0.47, 7.97)
> 34 years	219(81.1)	51(18.9	0.90(0.39, 2.08)	5.49(1.13, 26.65)*
Marital form of respondent’s husband				
Monogamous	305 (76.6)	93 (24.4)	ref	ref
Polygamous	129 (86.0)	21 (14.0)	0.53 (0.32, 0.90)	0.62(0.27, 1.42)
Possession of radio				
No	297 (86.3)	47 (13.7)	ref	Ref
Yes	138 (67.3)	67 (32.7)	3.07 (2.01, 4.69)	4.42(1.75, 11.20)*
Desire time for another child				
Within 2 year	50 (92.6)	4 (7.4)	ref	ref
After 2 years	278 (74.9)	93 (25.1)	4.18(1.47, 11.89)	7.13(1.53, 33.30)*
No desire	106 (86.2)	17 (13.8)	2.01(0.64, 6.27)	6.34(1.13, 35.67)*
Perceived cultural acceptability				
No/not sure	259 (84.6)	47 (15.4)	ref	ref
Yes	176 (72.4)	67 (27.6)	2.098(1.38, 3.19)	2.10 (1.09, 4.03)*
Perceived socio-economic benefits				
No/not sure	156 (88.6)	20 (11.4)	ref	ref
Yes	279 (74.8)	94 (25.2)	2.63(1.56, 4.42)	0.80(0.33, 1.94)
Couple discussion				
Never discussed	362 (94.3)	22 (5.7)	Ref	ref
Discussed	73 (44.2)	92 (55.8)	20.74(12.22,35.2)	4.63(2.15, 9.98)**
Perceived husband awareness				
No/not sure	223 (95.3)	11 (4.7)	Ref	ref
Yes	212 (67.3)	103 (32.7)	9.85 (5.14, 18.86)	1.18(0.45, 3.12)
Perceived Husband approval				
No or not sure	356 (93.9)	23 (6.1)	Ref	ref
Yes	76 (45.5)	91 (54.5)	18.5(11.02, 31.2)	8.0(3.52, 18.19)**
Proximity to HF				
< =15min	232 (74.1)	81 (25.9)	3.29(1.45, 7.47)	2.26(0.66, 7.78)
16–30 min	137 (84.0)	26 (16.0)	1.79(0.74, 4.334)	0.84(0.21, 3.33)
> 30 min	66 (90.4)	7 (9.6)	ref	ref
Visited by Health extension workers in the last 12 months				
Not visited BY HEW	159 (91.1)	14 (8.1)	ref	ref
Visited by HEW	276 (73.4)	100 (26.6)	4.12(2.28, 7.441)	0.75(0.19, 3.85)
Discussed with Health extension workers in the last 12 months				
Not discussed	216 (90.8)	22 (9.2)	ref	ref
Discussed	216 (70.1)	92 (29.9)	4.18 (2.53, 6.91)	5.99(1.81, 19.85)*
Socio Economic Status (SES) index				
Very lower	100(87.0)	15(13.0)	ref	Ref
Lower	94(87.0)	14(13.0)	0.99 (0.46, 2.17)	0.65(0.20, 2.16)
Middle	83(80.6)	20(19.4)	1.61(0.77, 3.33)	1.58(0.49, 5.10)
Highest	77(68.1)	36(31.9)	3.12 (1.59, 6.10)	0.42(0.11, 1.54)
Very higher	81(73.6)	29(26.4)	2.39 (1.20, 4.75)	0.53(0.14, 2.03)

*p < 0.05, **p < 0.001

## Discussion

The current modern contraceptive utilization rate by pastoralist women in the Bale Eco-region was by half lower than the national and regional averages of (42%) and (39.1%) respectively [7]. And it was also far lower when compared to the (69.5%) and (66.2%) modern contraceptive utilization rates reported in Southern and Northern parts of the country respectively [13, 14]. However, the utilization rate was better when compared to the (6.9%) MCU reported from Afar pastoralist region in Ethiopia [18]. The less utilization of the physically available family planning service by pastoralist women could be due to the influence of oppositions and uncertainty about the religious and cultural acceptability of the methods.

Among the total modern contraceptive utilizers, about (92.1%) relayed on 3 short-acting contraceptive methods which include: (78.1%) injectable method, (9.6%) oral contraceptive pills, and (4.4%) lactational amenorrhea method; while only (7.9%) of the total contraceptive users rely on implants. The current finding on the modern contraceptive method mix in the study area was in line with study findings reported from Rwanda where (64.1%) of the contraceptive users relayed on short-acting contraceptive methods and it also agrees with the findings reported from Southern Ethiopia where (57.0%) of women were predominantly using Short-acting contraceptive methods [13, 19]. While it was in the contrary to the study findings that reported (16.8%) and (6.6%) IUDs utilization in central Ethiopia [13, 20] the current study not identified users of the more effective and long-acting modern contraceptive method like the IUDs and Sterilization (surgical) method by pastoralist women in the Bale Eco-region. This implies that women who decided to use contraceptive method are not using the more effective long-acting contraceptive methods which require less frequency of visits to the health facilities for subsequent doses. The predominant utilization of short-acting methods could be due to the service provider influence on the users made them select the short-acting methods which are provided with a lesser level of provider skill and expertise. Therefore the Ministry of health should strengthen the skill of the service providers in the provision of quality family planning counselling and service and further diversify the method mix to enhance the use of more effective modern contraceptive methods by pastoralist women.

The findings of our study revealed wide-spread traditional practice of early marriage by women and polygamy (the practice of having more than one wife) among men. In the study area (73.7%) of the women were married the first time before the age of 18 years and (27.2%) of the respondents reported that their husband has more than one wife (polygynous). Early marriage has a remarkable effect on childbearing because women who marry early have on average a longer period of exposure to the risk of pregnancy and give birth to a greater number of children over their lifetimes. Similarly, Polygyny has implications for coital frequency and, therefore, fertility. Both early marriage and polygynous union in the study area were more than the national rural levels of (16.5 years) mean age at first marriage and (12%) of polygynous union reported in the 2011 Ethiopian demographic and health survey [6], this also agree with findings from Afar regions, Ethiopia that revealed (95%) of early marriage by pastoralist women [21]. The women age at their first marriage was against the Ethiopian Revised Family Code Proclamation, which declares that the minimum age at first marriage is 18 for both sexes [22]. Therefore it demands the government of Ethiopia should enforce the family law to increase the age at first marriage and polygynous marriage particularly in pastoralist communities such as the Bale eco-region.

Although (95.3%) of the respondents were familiar (able to spontaneously mention) at least one modern contraceptive method, the study revealed the presence of devastating perception towards religious and cultural acceptability of contraceptive use by women in the area. More than nine Women in ten (92.4%) believe that the use of FP is not supported by religious leaders and (55.7%) of them had a concern about the societal acceptability of the use of modern contraceptive methods. Such large proportion of unfavourable perceptions of women in one or another way could hamper usage of contraception in the rural areas and this is also supported by scholars from Muslim countries who correlate low contraceptive use with the level of isolation, poverty, illiteracy, and to a large extent, religious misinterpretations/misconceptions [23].

This study determined that, even though two third (67.7%) of the pastoralist women want to space or postpone the next childbirth for at least 2 years and another (22.3%) of the women desire to limit the number of their children, they are not actual utilizing the methods and this resulted in disproportionately lower level of family planning demand satisfied by pastoralist women. And this is in line with previous studies that reported a large number of couples who desired FP services was not receiving the service for a variety of reasons including religious oppositions, especially in the rural remote areas where the media is still not reaching and influencing mindset [4, 21, 23]. Therefore oppositions either due to religious beliefs, or concern of societal objection could be one of the major reasons for less use of modern contraceptive methods by pastoralist women in the Bale eco-region despite the women’s fertility desire. Besides such large proportion of perceived opposition against modern contraceptive utilization in the study area could be due to respondent’s outright resistance that can stem from a woman’s wrong or correct perception towards the religious beliefs of her partner and the community’s views related to modern contraceptive use.

Women who discussed family planning with their partner (couples family planning discussion) during the last 12 months period was one of the factors that determine the use of modern contraceptive methods. And this finding coincides with the findings reported by various other researchers from different community contexts who frequently reported discussion among couple was decisive to women’s contraceptive use [16, 17, 19, 24, 25]. And this could be due to either both women and men need encouragement from partners to apply their knowledge to their own situation or women and men who were reluctant to apply their understanding may need support from someone who had a close relationship with them. Apart from couple discussion, pastoralist women perception about their husband’s approval of FP use had an independent effect (*p* < 0.001) on their modern contraceptives service utilization. This is also supported by other studies conducted in Nigeria and elsewhere in Ethiopia which reported husband’s approval of a contraceptive method use had a significant impact on the ever use of modern contraceptives by women [26–29]. The possible reason might be husband’s approval and consent which motivates the women to make a decision towards modern contraceptive utilization. Therefore this could have an implication on the importance of promoting couple discussion and the active involvement of husbands in family planning education and counselling will encourage a couple to make an informed choice and decisions together.

Furthermore family planning discussions with Health Extension Workers during a 12 months period was also an important predictor of contraceptive use by women in our study area, which was also previously reported by other researchers in Ethiopia that concluded women who discussed issues of contraceptive use and counselled by health service providers were more likely to use the service [30–33]. This could be due to the discussion may clarify misconceptions and concerns related to modern contraceptive methods and could be encouraged and supported by the HEW to choose the method that best matches to their need. Therefore emphasis should be made to the importance of HEW’s scheduled group discussion sessions for pastoralist women in a fixed place within the village or through house to house visits to expand the possibility of supporting pastoralist women who are vulnerable and less likely to seek the service by themselves unless otherwise encouraged and supported by health service providers and significant others like friends and husbands.

Besides what has been discussed so far, the perception of the respondents about the community’s opinion towards family planning and birth spacing was the other factor which predicts modern contraceptive utilization (*p* < 0.05). This finding is also in line with other studies that reported objections to family planning by husbands, in-laws and other community members significantly affects women’s behaviour related to contraceptive use [34, 35]. Hence those pastoralist women who don’t perceive modern contraceptives use is culturally accepted are less likely to freely decide and use it, due to the fact that cultural context is central to the response to health and wellbeing [36], the pastoralist women could be influenced more by their perception of their social network’s approval of family planning than by their own approval [37]. Furthermore the oppositions from husbands and the other community members that stems from the community’s expectations of married women to give birth for many children and the long-held custom of large family sizes by parents, and grand-parents in pastoralist community, entrenched with moral and religious beliefs seriously hampers the women’s right to freely choose and utilize modern contraceptive methods. Therefore, in order to ensure the rights of all citizens access voluntary family planning the reproductive health and family planning programs in pastoralist districts should target the cultural and social relationships and should be tailored and actively involve pastoralist women, their husbands, religious leaders and significant others.

Consequently, this calls for vigorous behavioural change campaigns and advocacies using different routes to liquefy the unfavourable sociocultural barriers towards family planning in general and modern contraceptive methods in particular in remote pastoralist communities. Important shifts in program priorities and the emergence of strong local leadership will be needed to legitimize the idea of smaller families and contraception in communities where population pressure and high fertility rate could threats biodiversity hotspots like in the Bale eco region.

Although the respondents were asked and reported past experiences and alike other cross-sectional studies there is a potential liability to recall bias; the investigators believed that the mentioned limitations can’t inflict major impact on the validity of study findings and the findings could contribute to the design of appropriate family planning demand generation intervention in vulnerable community groups where less attention was previously given.

## Conclusions

The study identified lower modern contraceptive method utilization by pastoralist women in the study area, and the majority of the contraceptive users rely on short-acting contraceptive methods. Early marriage by women and polygynous marriage by men were common traditional practices in the study population. The presence of devastating perceptions towards religious and societal acceptability of modern contraceptive use by women could be the major reasons for lesser utilization of the service. Couple discussion, husband’s approval, perceived cultural acceptability and discussion with HEW were the factors associated with modern contraceptive utilization by pastoralist women. Family planning programs should target cultural and social relationships to tailor and actively involve pastoralist women, husbands, religious leaders and significant others in pastoralist community.

## Additional file

Additional file 1: The SPSS datasets used or analysed during the current study. (SAV 175 kb)

## Abbreviations

AOR: Adjusted odds ratio
BER: Bale Eco Region
BMNP: Bale mountains national park
CPR: Contraceptive prevalence rate
CSA: Central statistical agency
EDHS: Ethiopian demographic and health survey
FDRE: Federal Democratic Republic of Ethiopia
FGAE: Family Guidance Association Ethiopia
FMOH: Federal ministry of health
FP: Family planning
HEP: Health extension package
HEW: Health extension workers
HMIS: Health management information system
ICFP: International conference on family planning
ILAPMS: Injectable long acting and permanent methods
IUDs: Intra uterine devices
LAM: Lactational amenorrhea method
MOH: Ministry of health
PCA: Principal component analysis
RCSD: Research and community service directorate
SHARE: Support for horn of Africa resilience
SNNPR: Southern Nations Nationalities and Peoples Region
TFR: Total fertility rate
UNFPA: United Nations Fund for Population Affairs
WHO: World Health Organization
